# Processing light with an optically tunable mechanical memory

**DOI:** 10.1038/s41467-021-20899-w

**Published:** 2021-01-28

**Authors:** David P. Lake, Matthew Mitchell, Denis D. Sukachev, Paul E. Barclay

**Affiliations:** Department of Physics and Astronomy and Institute for Quantum Science and Technology, University of Calgary, Calgary, AB Canada

**Keywords:** Microresonators, Optical properties of diamond, Nanophotonics and plasmonics

## Abstract

Mechanical systems are one of the promising platforms for classical and quantum information processing and are already widely-used in electronics and photonics. Cavity optomechanics offers many new possibilities for information processing using mechanical degrees of freedom; one of them is storing optical signals in long-lived mechanical vibrations by means of optomechanically induced transparency. However, the memory storage time is limited by intrinsic mechanical dissipation. More over, in-situ control and manipulation of the stored signals processing has not been demonstrated. Here, we address both of these limitations using a multi-mode cavity optomechanical memory. An additional optical field coupled to the memory modifies its dynamics through time-varying parametric feedback. We demonstrate that this can extend the memory decay time by an order of magnitude, decrease its effective mechanical dissipation rate by two orders of magnitude, and deterministically shift the phase of a stored field by over 2*π*. This further expands the information processing toolkit provided by cavity optomechanics.

## Abstract (web summary)

The use of cavity optomechanics for optical information processing is hindered by mechanical dissipation and the difficulties in stored signal control. Here, the authors improve performances on both fronts using time-varying parametric feedback through an additional optical field in a multi-mode cavity.

## Introduction

Information processing devices exhibit dissipation due to their coupling to external degrees of freedom. This permits energy to leave and fluctuations to enter the system^1^, and typically degrades the performance of components such as memories. Within the field of optomechanics^2,3^, this has motivated tremendous progress in reducing intrinsic dissipation through precise tailoring of device geometry and materials^4–7^. A complementary approach, sometimes referred to as reservoir engineering^8^, uses dissipation channels to enhance system properties, typically via an auxiliary coherent source that couples to the system. Here, we show that when reservoir engineering is generalized to incorporate dynamic control of this external coupling, a system’s steady state can be adiabatically manipulated. By dynamically varying the coupling of an optomechanical memory to an auxiliary control field–a reservoir mode–we demonstrate that stored light can be coherently modified. This is a crucial step towards realizing the optical processing of light stored in the mechanical motion of a nanoscale device.

The concept of using an external field to manipulate optical information via coupling to a mechanical degree of freedom has been previously explored in Brillouin scattering optomechanics^9^, where powerful functionality including pulse storage^10,11^, all optical signal processing^12^, and coherently refreshed memory^13^ have been demonstrated in cm-long waveguides. Cavity optomechanical devices, in which optical and mechanical modes can be confined and spatially overlapped within wavelength-scale volumes, allow the nearly complete transfer of an optical excitation to low dissipation mechanical resonances. These devices operate with relatively low power and occupy micron-scale footprints, and have been used for information storage^14,15^. However, manipulating this stored information has not been demonstrated.

In this work, we use a diamond optomechanical microdisk cavity to realize a memory based on optomechanically induced transparency^16,17^ that converts a weak optical input to a long-lived mechanical excitation. We then show that by adjusting the frequency of a field input to a reservoir mode, we can effectively reduce the mechanical dissipation of the device through parametric feedback until it is just below the self-oscillation threshold, allowing the memory lifetime to be extended. Through precise tuning of the reservoir-field frequency, we directly observe a memory lifetime enhancement of over seven times, and realize an over 150 times reduction in the resonator’s effective mechanical dissipation rate. Finally, we demonstrate that by dynamically varying the reservoir-field frequency the phase of the stored signal can be manipulated.

## Results

Diamond microdisks can operate as multimode cavity optomechanical systems whose optical whispering gallery modes are coupled by radiation pressure to the motion of the device’s mechanical resonances^18^. Coherent multimode optomechanical coupling is possible in these devices even at room-temperature and ambient conditions^19^ thanks to the low-energy dissipation rates, *κ* and Γ, of their optical and mechanical modes, respectively, combined with diamond’s ability to support high-intensity fields without suffering from nonlinear absorption and heating. This latter property increases the maximum number (*n*) of photons that can be supported by the microdisk, which enhances the photon-assisted optomechanical coupling rate $g=\sqrt{n}{g}_{0}$. These devices have a high single-photon optomechanical coupling rate *g*_0_ due to their wavelength-scale dimensions and strong spatial overlap of the microdisk’s whispering gallery modes with its mechanical radial breathing mode.

The condition for coherent optomechanical coupling in both classical and quantum devices is cooperativity *C* = 4*g*^2^/*κ*Γ > 1, and can be achieved simultaneously by using multiple modes of a diamond microdisk. Multimode cavity optomechanics enables optical wavelength conversion^20–23^, photon entanglement^24,25^, and low-noise frequency conversion in the microwave domain^26^. We show in this paper that multimode diamond microdisks are excellent platforms for implementing memories whose stored information can be manipulated via dynamic reservoir engineering^27,28^.

**Fig. 1:**
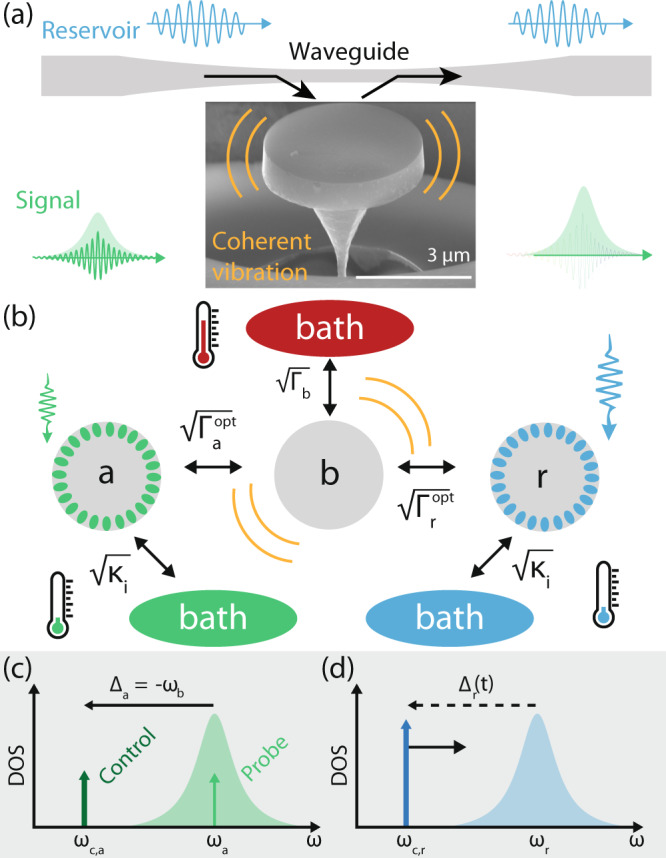
Key elements of the tunable optomechanical memory. **a** Annotated scanning electron micrograph of the diamond microdisk optomechanical cavity. A tapered optical fiber is utilized to couple light into two of the microdisk whispering gallery modes which in turn are coupled to mechanical vibrations of the radial breathing mode of the microdisk. **b** Schematic of the system under study where the mechanical mode *b* is coupled to the environment (red bath), an optical reservoir mode, *r*, and the optical mode *a* used for writing and reading the information, at the indicated rates. While coupling of the mechanical mode to the surrounding environment is an intrinsic property of the device and environment, the coupling to *r* may be manipulated through the use of a control laser. **c**, **d** Density of states (DOS) picture showing the detuning of the control and probe laser fields for (**c**) mode *a* and (**d**) mode *r*. Here, *ω*_c,a_ and *ω*_c,r_ are the frequencies of the control lasers for the signal and reservoir modes, respectively.

The system used in this work is illustrated schematically in Fig. 1. Information input to an optical “signal” mode (*a*) of a diamond microdisk cavity is transferred via optomechanical coupling to the device’s mechanical mode (*b*). Simultaneously, the mechanical mode dynamics are modified through its coupling to an optical “reservoir” mode (*r*). The optical and mechanical modes are characterized by their frequencies (*ω*_a,r_, *ω*_b_) and energy decay rates (*κ*_a,r_, Γ_b_), respectively. The reservoir mode is driven by a control laser whose detuning from resonance, Δ_r_, sets the phase lag of its optomechanical coupling to the mechanical resonator, and whose power, *P*_r_ (defined here as the power input to the fiber taper waveguide), sets the coupling strength. This tunable resonator–reservoir interaction induces additional mechanical dissipation ${{{\Gamma }}}_{{\rm{r}}}^{{\rm{opt}}}$ and shifts the mechanical resonator frequency by ${\omega }_{{\rm{r}}}^{{\rm{opt}}}$, two effects widely studied in single-mode optomechanical systems, for example in demonstration of mechanical ground-state cooling^29,30^.

To process information stored in the mechanical resonator, we dynamically adjust the power and detuning of the reservoir input field. The resonator evolution is governed by (Supplementary Note 1):

1 $${\dot{\hat{b}}}= \,-\!\left(i{\omega }_{{\rm{b}}}^{{\rm{eff}}}({{{\Delta }}}_{r},{g}_{r})+\frac{{{{\Gamma }}}_{{\rm{b}}}^{{\rm{eff}}}({{{\Delta }}}_{r},{g}_{r})}{2}\right)\hat{b}+\sqrt{{{{\Gamma }}}_{{\rm{b}}}}{\hat{e}}_{{\rm{in}}}\\ \;+\,{g}_{{\rm{r}}}\sqrt{{\kappa }_{{\rm{r}}}}{\chi }_{{\rm{r}}}({\omega }_{{\rm{b}}};{{{\Delta }}}_{r}){\hat{r}}_{{\rm{in}}}+{g}_{{\rm{r}}}\sqrt{{\kappa }_{{\rm{r}}}}{\chi }_{{{\rm{r}}}^{\dagger }}({\omega }_{{\rm{b}}};{{{\Delta }}}_{r}){\hat{r}}_{{\rm{in}}}^{\dagger },$$

where $\hat{b}$ is the phonon annihilation operator, ${\hat{e}}_{{\rm{in}}}$ is the thermal bath input field, ${\hat{r}}_{{\rm{in}}}$ is the optical reservoir input field, ${g}_{{\rm{r}}}\propto \sqrt{{P}_{r}}$ is the photon-assisted optomechanical coupling rate between the reservoir mode and the resonator, and *χ*_r_ is the reservoir mode’s optical response in the frame of the reservoir mode control laser. The key feature that we test and exploit is the ability to dynamically control the memory’s effective mechanical frequency, ${\omega }_{{\rm{b}}}^{{\rm{eff}}}={\omega }_{{\rm{b}}}+{\omega }_{{\rm{r}}}^{{\rm{opt}}}(t)$, and effective damping rate, ${{{\Gamma }}}_{{\rm{b}}}^{{\rm{eff}}}={{{\Gamma }}}_{{\rm{b}}}+{{{\Gamma }}}_{{\rm{r}}}^{{\rm{opt}}}(t)$, by adjusting the reservoir-mode parameters *g*_*r*_(*t*) and Δ_*r*_(*t*) as a function of time. Notably, we find that when we input a field to mode *a* to this system, the memory operates as if it is a conventional single-mode cavity optomechanical device whose mechanical resonator dynamics have been renormalized. This regime is valid if Γ_b_ ≪ *κ*_r_.

Equation (1) shows that the reservoir-mode coupling acts as a dissipative process, and not a direct drive. Since we alter the effective dissipation of the system, we use the descriptor reservoir engineering, in analogy to the work that introduced this term^8^. Note that although the effects demonstrated below can be described classically, the quantum formalism used here allows the analysis of noise processes, including photon-phonon scattering, that will ultimately limit memory performance.

Below we test the validity of this description and demonstrate applications of dynamic reservoir coupling through three experiments. First, we show an enhancement of the system’s optomechanical cooperativity due to its renormalized mechanical dissipation and switch the dynamics of the system from overall loss to the overall gain. Second, we increase the optomechanical memory storage time through control of ${{{\Gamma }}}_{{\rm{b}}}^{{\rm{eff}}}$. Finally, we control the phase of a stored mechanical signal through manipulation of ${\omega }_{{\rm{b}}}^{{\rm{opt}}}$ as a function of time.

### Engineering the system dynamics

**Fig. 2:**
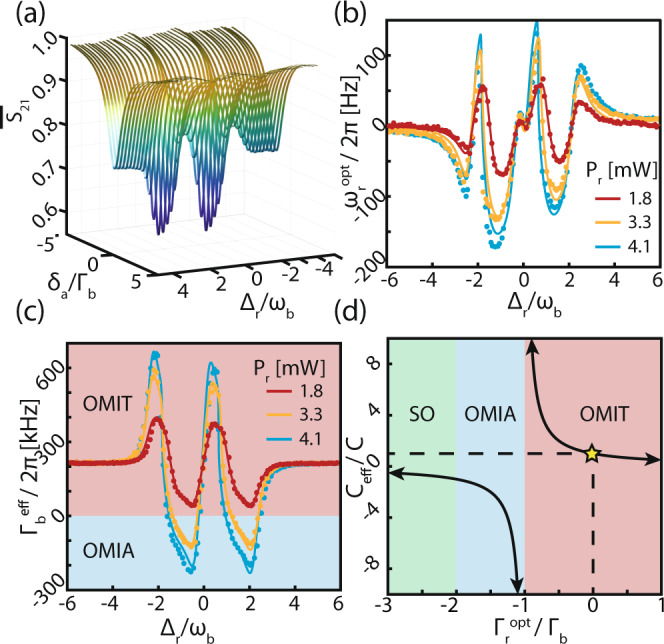
Tuning the mechanical resonator dynamics. **a** Normalized OMIT scans as a function of mode *a* probe-cavity detuning, *δ*_a_, and control-reservoir detuning, Δ_r_, and *S*_21_ is the probe laser reflection measured using a vector network analyzer. The changes in the transparency window as a function of Δ_r_ are indicative of reservoir interactions. Optomechanically manipulated effective (**b**) mechanical frequency and (**c**) damping as a function of Δ_r_ and the reservoir-mode input power, *P*_r_. (**d**) Illustration of effective cooperativity, *C*_eff_, for varying ${{{\Gamma }}}_{{\rm{b}}}^{{\rm{eff}}}$ controlled by the reservoir mode, when ${{{\Gamma }}}_{{\rm{a}}}^{{\rm{opt}}}={{{\Gamma }}}_{{\rm{b}}}$ due to the presence of the mode *a* control field. Three different regimes of operation are shown: OMIT OMIA, and self-oscillation (SO). The yellow star indicates the operating point when the reservoir field is off.

We first probe how the dynamics of the mechanical resonator, and its resulting coupling to light, are affected by the resonator–reservoir interaction. This is accomplished using optomechanically induced transparency (OMIT) spectroscopy^16,17^. OMIT creates a transparency window in the cavity lineshape whose properties depend on the dynamics of the optomechanical system. By coherently coupling a probe field in *a* to the mechanical resonator for varying reservoir control laser settings, we can learn about the influence of the reservoir on the resonator. These measurements are shown in Fig. 2a, which were obtained using a fiber taper waveguide to evanescently couple the reservoir control laser to mode *r* (*ω*_r_/2*π* = 192 THz, *κ*_r_/2*π* = 1.13 GHz) for varying Δ_r_, while performing OMIT spectroscopy on mode *a* (*ω*_a_/2*π* = 197 THz, *κ*_a_/2*π* = 0.856 GHz) using a weak resonant probe laser and a control laser red detuned by *ω*_b_. Note that in all measurements presented below the probe field is generated by modulating the control laser and is typically in the *μ*W range (see “Methods”). The device used here has *ω*_b_/2*π* ~ 2.14 GHz and Γ_b_/2*π* = 190 kHz for the radial breathing mode and operates in the resolved sideband regime for modes *a* and *r*(*ω*_b_/*κ*_a_ = 2.5, *ω*_b_/*κ*_r_ = 1.9). Coupling to other mechanical modes of the microdisk was not observed. Note that the optical reservoir mode is a standing wave doublet (see “Methods” and Supplementary Note 1) formed from backscattering in the microdisk^31,32^, whose most apparent effect is the two sets of minima and maxima in Fig. 2a. In all measurements, the mode *a* control field detuning was set relative to the lowest frequency doublet resonance.

This measurement was repeated for three different values of *P*_r_, with the probe control laser fixed at input power *P*_a_ ~ 0.26 mW (intracavity photon number *n*_a_ ~ 3.2 × 10^4^). At each reservoir setting ${{{\Gamma }}}_{{\rm{b}}}^{{\rm{eff}}}$ and ${\omega }_{{\rm{b}}}^{{\rm{eff}}}$ were extracted from the OMIT window shape (see Supplementary Note 2), and are plotted along with fits to the data in Fig. 2b, c. The fits, which show excellent agreement with measurements, were obtained with *g*_r_ as the only fitting parameter. This confirms that optomechanical renormalization of the mechanical resonator dynamics by the reservoir field affect mode *a*’s optomechanical response as if ${{{\Gamma }}}_{{\rm{b}}}^{{\rm{eff}}}$ and ${\omega }_{{\rm{b}}}^{{\rm{eff}}}$ were the mechanical resonator’s intrinsic mechanical properties.

The system dynamics are most dramatically affected when ${{{\Gamma }}}_{{\rm{b}}}^{{\rm{eff}}}$ approaches zero and becomes negative. In a conventional OMIT system, the depth and width of the transparency window is parameterized by the optomechanical cooperativity, $C=\frac{4| {g}_{{\rm{a}}}{| }^{2}}{{\kappa }_{{\rm{a}}}{{{\Gamma }}}_{{\rm{b}}}}={n}_{\text{a}}\frac{4| {g}_{0}{| }^{2}}{{\kappa }_{{\rm{a}}}{{{\Gamma }}}_{{\rm{b}}}}$, where *g*_a_ is the photon-assisted optomechanical coupling rate for mode *a*. For the microdisk used here, *g*_0_/2*π* ~ 25 kHz^23^. Note that the optomechanical cooperativity differs from the single-photon cooperativity by a factor of *n*_a_. However, in our multimode system, OMIT is governed by an effective cooperativity ${C}_{{\rm{eff}}}=C\times {{{\Gamma }}}_{{\rm{b}}}/{{{\Gamma }}}_{{\rm{b}}}^{{\rm{eff}}}$. In the measurement presented here, we achieve a maximum *C*_eff_ = 83, which represents an enhancement of 158 × the bare *C* = 0.52 experienced by the mode *a* probe in absence of the reservoir field. This allows our system to act as though it has large cooperativity, enabling large light delays and narrow transparency windows (see Supplementary Note 2). This enhancement is limited only by how close to zero ${{{\Gamma }}}_{{\rm{b}}}^{{\rm{eff}}}$ can be tuned through adjustment of Δ_r_. Hence, it is not limited by available laser power since the regime of ${{{\Gamma }}}_{{\rm{b}}}^{{\rm{eff}}}<0$ can be reached for the powers used here (see below).

As ${{{\Gamma }}}_{{\rm{b}}}^{{\rm{eff}}}$ becomes negative, the mechanical resonator motion changes from experiencing an overall loss to an overall gain. Consequently, by adjusting our reservoir coupling, we are able to tune the system dynamics between OMIT and the regime of optomechanically induced amplification (OMIA). This gain would normally cause optomechanically induced self-oscillation to occur^33,34^. However, this instability is repressed in our multimode measurements by the optomechanical damping ${{{\Gamma }}}_{{\rm{a}}}^{{\rm{opt}}}$ induced by mode *a*’s OMIT process, provided ${{{\Gamma }}}_{{\rm{a}}}^{{\rm{opt}}}+{{{\Gamma }}}_{{\rm{b}}}^{{\rm{eff}}}\,> \,0$. The ability of the reservoir mode to tune the system dynamics between OMIT, OMIA, and self-oscillation regimes is illustrated in Fig. 2d for the special case that ${{{\Gamma }}}_{{\rm{a}}}^{{\rm{opt}}}={{{\Gamma }}}_{{\rm{b}}}$. In our measurements, the OMIA regime was entered for both the *P*_r_ = 3.3 mW and *P*_r_ = 4.1 mW settings when the control laser was blue detuned from the reservoir mode’s lowest frequency doublet feature by approximately *ω*_b_. This regime was not accessed during the storage measurements described in the following sections, and can only be measured due to the damping provided by the mode *a* fields.

### Pulse-storage manipulation

Pioneering experiments with Λ-type atomic systems have demonstrated that a strong control field can dramatically alter the optical response of a material, including rendering otherwise opaque materials transparent^35^, enhancing nonlinear processes^36,37^, and slowing the group velocity of a pulse of light^38,39^. Furthermore, by dynamically altering the transparency of a material, a pulse of light may be trapped and deterministically released at a later time^40–42^. Such schemes have been used to store light pulses for over a minute^43^, and have been proven to work at a single-photon level^44,45^.

Cavity optomechanical systems have similar capabilities. In OMIT, the interaction between optical mode *a* and the mechanical resonator is described by the beamsplitter Hamiltonian $${\hat{H}}_{\text{bs}}=\hslash {g}_{\text{a}}({\hat{a}}^{\dagger }\hat{b}+\hat{a}{\hat{b}}^{\dagger })$$ when the mode *a* control field is red detuned by *ω*_b_ from resonance^2^. Here, $$\hat{a}$$$$\left({\hat{a}}^{\dagger }\right)$$ and $$\hat{b}$$$$({\hat{b}}^{\dagger })$$ are annihilation (creation) operators for optical mode *a* and mechanical resonator mode *b*, respectively. This Hamiltonian allows for coherent exchange of excitations between modes *a* and *b*. By adjusting the control field amplitude, which in turn controls *g*_a_, a field input to *a* can be coherently and reversibly stored in the mechanical resonator^14,15,46,47^.

**Fig. 3:**
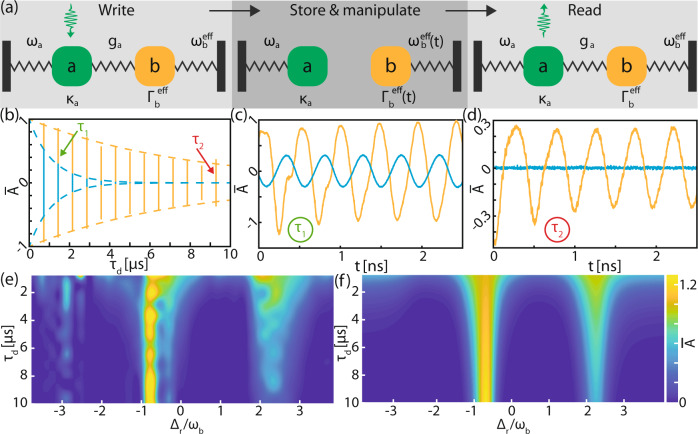
Enhancing the optomechanical pulse-storage time. **a** Outline of the storage protocol where the optomechanical coupling between optical mode *a* and the mechanical mode facilitates storage of an optical pulse as a mechanical excitation. Mode *r* is used to manipulate the mechanical damping rate, ${{{\Gamma }}}_{{\rm{b}}}^{{\rm{eff}}}$(t), and frequency, ${\omega }_{{\rm{b}}}^{{\rm{eff}}}$(t) which can be carried out concurrently with the storage process. **b** Normalized read pulse amplitude, $\bar{A}$ for the off-resonant, Δ_r_ ≫ *ω*_m_, case (cyan) and for Δ_r_ ~ *ω*_m_ (orange) as a function of *τ*_d_. These are fit to an exponential decay (dashed lines) to extract ${{{\Gamma }}}_{\,\text{b}}^{\text{eff}\,}$. **c**, **d** Zoom-in of of the read data shown in (**b**) for *τ*_d_ = (0.14, 0.93) μs, illustrating the enhancement in $\bar{A}$ when Δ_r_ ~ *ω*_m_. **e** Complete data set from which (**b**) was taken showing both an enhancement and reduction in storage time as a function of Δ_r_, which is compared to the expected behavior (**f**).

Here, we show that an optical field stored in the motion of an optomechanical memory can be dynamically modified by varying the resonator’s coupling to a reservoir mode. Our memory protocol is illustrated using mass-on-spring systems in Fig. 3a. During the writing stage, the red-detuned mode *a* control laser couples a weak signal field resonant with mode $\hat{a}$ to mode $\hat{b}$ at rate *g*_a_. Following the write stage, the control laser is removed, decoupling modes *a* and *b*. Finally, during the read stage, the reservoir control laser is removed and the signal control laser is turned back on, reconverting the stored mechanical signal to the optical domain. Our scheme deviates from a conventional optomechanical memory^15,46,47^ in two ways: not only does coupling to the reservoir modify the mechanical resonator dynamics as described above, the reservoir coupling can also be varied temporally. This both modifies the stored information and the rate at which it dissipates.

Through continuous amplification of the stored mechanical signal by the reservoir mode, the pulse-storage lifetime of the mechanical resonator can be extended. This is in a similar spirit to previous demonstrations of optomechanical amplification in waveguides^13^. In cavity optomechanics, amplification is achieved by setting Δ_r_ to +*ω*_b_, so that the reservoir-resonator interaction is governed by the parametric amplifier Hamiltonian $${\hat{H}}_{\text{amp}}=\hslash {g}_{\text{r}}(\hat{r}\hat{b}+{\hat{r}}^{\dagger }{\hat{b}}^{\dagger })$$, where $$\hat{r}$$$$({\hat{r}}^{\dagger })$$ is the annihilation (creation) operator for optical mode *r*. In the experiments, Δ_r_ is tuned until $${{{\Gamma }}}_{{\rm{b}}}^{{\rm{eff}}}$$ nearly vanishes, as in Fig. 2c. Note that in contrast to Brillouin scattering, the mechanical mode amplitude can grow exponentially during this interaction^2,48^.

To measure the storage time for a given reservoir setting, we varied the delay *τ*_d_ between the write and read pulses. The measured amplitude output during the read pulse encodes the temporal envelope of the mechanical signal, which decays exponentially at a rate ${{{\Gamma }}}_{{\rm{b}}}^{{\rm{eff}}}$. An example of this decay is plotted in Fig. 3b for two cases: when the reservoir control is optimally detuned for enhanced storage time (Δ_r_ ≈ −*ω*_b_ and ${{{\Gamma }}}_{{\rm{b}}}^{{\rm{eff}}}\ll {{{\Gamma }}}_{{\rm{b}}}$), and when the reservoir is far detuned (Δ_r_ ≫ *ω*_b_ and ${{{\Gamma }}}_{{\rm{b}}}^{{\rm{eff}}} \sim {{{\Gamma }}}_{{\rm{b}}}$).

Comparing the observed decay rates for each configuration indicates a 7 × enhancement in 1/*e* time from 1.1 μs to 7.7 μs due to the reservoir coupling. Examples of the signal extracted at two values of *τ*_d_ are shown in Fig. 3c, d, demonstrating the dramatic increase in the amplitude of the enhanced signal at longer timescales. Full measurement of the readout amplitude decay for varying Δ_r_ was also acquired, the results of which are plotted in Fig. 3e. This shows qualitative agreement to the theoretical amplitudes obtained from an analytic fit of ${{{\Gamma }}}_{{\rm{b}}}^{{\rm{eff}}}({{{\Delta }}}_{{\rm{r}}})$ and plotted in Fig. 3f. Note the doublet nature of the reservoir mode is again evident in the measurement. Differences between theory and experiment are understood to be a consequence of thermal drifts in the resonance frequencies of the modes, as well as wavelength drifts in the readout laser used in the experiment. These imperfections lead to a deviation of the readout signal from a sinusoid in Fig. 3c and will reduce the fidelity of the reamplified memory. They can be mitigated by an active stabilization of Δ_*r*_ but further measurements with shaped input pulses are required to fully characterize their effect.

The optomechanical memory’s storage time depends on dephasing governed by $1/{{{\Gamma }}}_{\,\text{b}}^{\text{eff}\,}$ and on added noise. The latter is created by the optical field, which can amplify thermal phonons and spontaneously generate phonons, in addition to amplifying desirable signal phonons. Ignoring spontaneous processes, given a stored signal with an initial signal-to-noise ratio (SNR_0_) defined by the ratio of the signal and thermal phonon populations, for large SNR_0_ the thermal and signal phonon populations become equal at time ${t}_{s} \sim 1/{{{\Gamma }}}_{\,\text{b}}^{\text{eff}\,}\mathrm{ln}\,\left({\text{SNR}}_{0}\right)$. This storage time exceeds the 1/*e* decay time, as it is defined by the minimum acceptable SNR of the retrieved signal. It ignores the reduction to the initial thermal population from the OMIT write step, which will further extend *t*_*s*_; a more general expression is given together with an analysis of storage time in Supplementary Note 4. The maximum SNR_0_ is set by the device’s temperature combined with its maximum achievable oscillation amplitude. From previous measurements an SNR_0_ of 1000 at room temperature should be achievable^18^. Together with a realistic value of ${{{\Gamma }}}_{\,\text{b}}^{\text{eff}\,}=2\pi \times 1$ kHz, a memory time of ≈ 5 ms is predicted. Finally, note that the ultimate limit on storage time will be set by generation and amplification of Stokes phonons, which were not considered in this analysis but will become important when operating in the quantum regime.

We also emphasize that thermal phonons from the environment preclude storage of quantum states when operating at room temperature, due to the persistent thermal occupation of the memory. Even in the absence of thermal noise, phonons introduced by the reservoir laser, e.g., through spontaneous Stokes scattering, will degrade the device’s ability to store quantum states. These effects could potentially be mitigated by utilizing phononic shielding^29,49^, and by further increasing the optomechanical cooperativity through reducing the device’s optical loss (which is limited by surface roughness^50^), its mechanical dissipation, and its optical mode volume.

However, in the measurements of decay time presented here, technical noise dominated over amplified mechanical noise. In particular, drifts in control and probe laser detunings required operating at a lower *P*_r_ compared to the cooperativity enhancement measurements in Fig. 2, to ensure that the system did not enter the unstable regime (${{{\Gamma }}}_{\,\text{b}}^{\text{eff}\,}<\,0$). This lower power explains the modest observed decease in the decay time compared to the 158 × cooperativity enhancement reported above. By operating more closely to the self-oscillation threshold through stabilization of Δ_r_, reductions in decay time similar to or exceeding the cooperativity enhancement will be possible.

Another important characteristic of a memory is its time–bandwidth product (TBP). A conservative measure of this for our system can be obtained from the product of the 1/*e* decay time and the mechanical linewidth. For CW excitation of the reservoir, this product will not change with ${{{\Gamma }}}_{{\rm{b}}}^{{\rm{eff}}}$ as any increase in decay time will be accompanied by a reduction in linewidth; it is 1.38 for our device which is typical for OMIT-based memories^14,46^ and is an order of magnitude smaller than in Brillouin waveguide memories^13^. However, note that in waveguide systems storage time is often defined by SNR, as discussed above, resulting in a TBP $\propto \mathrm{ln}\,\left({\text{SNR}}_{0}\right)$ dependent on the initial signal strength. Increasing the bandwidth can be realized by using multiple mechanical modes or cascaded resonators^51^, which is predicted to provide a maximum bandwidth ~ Γ_*b*_*C*. Alternatively, by operating with a reservoir field that is turned on adiabatically during the storage phase, one could combine enhanced storage times and the intrinsic bandwidth of the mechanical resonator.

**Fig. 4:**
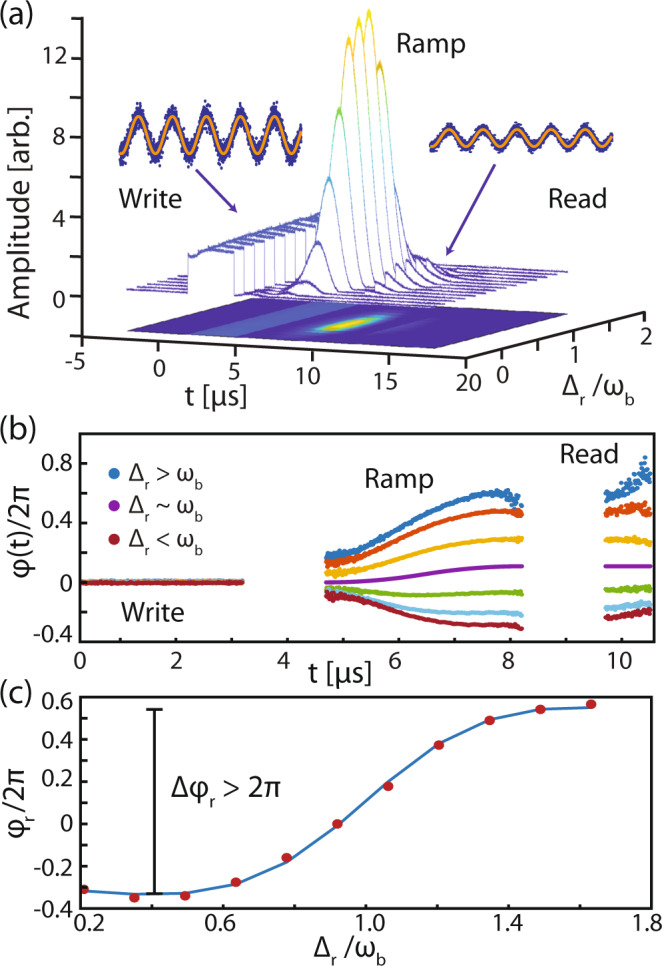
Tuning the phase of the retrieved pulse. **a** Amplitude of the acquired signals vs. time and reservoir detuning for the phase-manipulation experiment. In this experiment, no optical filtering was used, so that the write, ramp, and read pulses may all be detected. **b** Phase of the mechanical oscillator vs. time as inferred from the optical output of the device. **c** Final phase as a function of Δ_r_ along with a fit to the data based on the predicted temporally varying optical spring effect associated with the ramp pulse.

We next show that the phase of the stored pulse can be controlled by dynamically varying the reservoir-mode input. By changing ${\omega }_{{\rm{b}}}^{{\rm{eff}}}$ adiabatically and hence the frequency of the stored pulse, we can complete a trajectory that moves away from and then returns to the original frequency. Over the course of this trajectory, the mechanical oscillator acquires a dynamical phase $\varphi ({t}_{2})=\varphi ({t}_{1})+\mathop{\int}\nolimits_{{t}_{1}}^{{t}_{2}}\delta \omega (t)dt$, assuming that we return to the original mechanical frequency. This is analogous to a pendulum whose length is adjusted in time^52^. In our experiment, we varied the amplitude of the reservoir mode in time using an amplitude electro-optic modulator driven by a symmetric RF ramp pulse (see “Methods”) for various Δ_r_ as shown in Fig. 4a. Here, the ramp pulse was 3.5 μs long and was situated 1.5 μs after the write, and before the read pulse. By fitting the beat note detected at the fiber output for each of the write, ramp, and read pulse segments, we can plot the phase as referenced to the well-defined write pulse, as shown in shown in Fig. 4b. Here, we have removed the phase shift associated with the spring effect induced by the write pulse, which added a linear slope to the ramp and read pulse segments. This allows us to isolate the shift due to the spring effect associated with the reservoir-mode ramp pulse (see Supplementary Note 5). From the read pulse segments, we extract the phase relative to the write pulse, demonstrating a reservoir controlled phase shift, Δ*φ* > 2*π* as shown in Fig. 4c. Further studies of quantum noise generated by the reservoir mode during the frequency tuning process are required to assess the performance of this technique in the quantum domain.

## Discussion

In this work, we have demonstrated in-situ control of an optomechanical memory, bypassing the usual limitations imposed by the intrinsic damping rate and generating controllable phase shifts in the stored information. The diamond microdisk platform benefits from the diamond’s superior optical and mechanical properties. Low optical absorption, high thermal conductivity, and a cavity-based design minimize the necessary control and probe lasers powers, compared to waveguide optomechanics, by creating high intracavity light intensities. Low mechanical dissipation ensures that high effective cooperativity and vanishing effective mechanical dissipation can be achieved at the modest power levels used here, resulting in an increased memory lifetime that is primarily limited by the stability of the system not available optical power. Together with a small footprint, this allows for compact, fully integrated optical memories whose multimode nature allows stored information to be coherently manipulated.

In the future, the performance of the demonstrated optomechanical memory can be improved by cooling and cascading devices to enhance the memory time and memory-bandwidth product. By virtue of operating in the sideband resolved regime, ${{{\Gamma }}}_{{\rm{b}}}^{{\rm{eff}}}$ and ${\omega }_{{\rm{b}}}^{{\rm{eff}}}$ for our device are linearly independent of *g*_r_ and Δ_r_^53^. As a result, it should be possible to incorporate dynamical control of the damping rate, enabling pulse compression by the realization of a time lens^54,55^ (see Supplementary Note 6) as well as other processing functionality inspired by Brillouin scattering based signal processing^12^. Operating the device in the OMIA regime will allow demonstration of narrow spectral filters. Furthermore, spatially confined and optically-induced mechanical oscillations can be coupled, for example, to spin qubits and electromagnetic fields, facilitating the development of next-generation hybrid quantum systems.

## Methods

### Fabrication

The microdisks studied here were fabricated from an optical grade, chemical vapor deposition (CVD) grown 〈100〉-oriented single-crystal diamond substrate supplied by Element Six, according the process outlined in detail in ref. ^50^. The polished substrates were first cleaned in boiling piranha, and coated with ~400 nm of PECVD Si_3_N_4_ as a hard mask. To avoid charging effects during electron beam lithography (EBL), ~5 nm of Ti was deposited before the ZEP 520A EBL resist. The developed pattern was transferred to the hard mask via inductively coupled reactive ion etching (ICPRIE) with C_4_F_8_/SF_6_ chemistry. The anisotropic ICPRIE diamond etch was performed using O_2_, followed by deposition of ~250 nm of conformal PECVD Si_3_N_4_ as a sidewall protection layer. The bottom of the etch windows were then cleared of Si_3_N_4_ using a short ICPRIE etch with C_4_F_8_/SF_6_. This was followed by a zero RF power O_2_ RIE diamond undercut etch to partially release the devices. The undercutting process was interrupted and an ~100 nm layer of SiO_2_ is deposited via electron beam evaporation to alter the microdisk pedestal profile before finishing the undercut. Lastly, the Si_3_N_4_ layer was removed using a wet etch in 49% HF, and the devices were cleaned again in boiling piranha.

### Apparatus

**Fig. 5:**
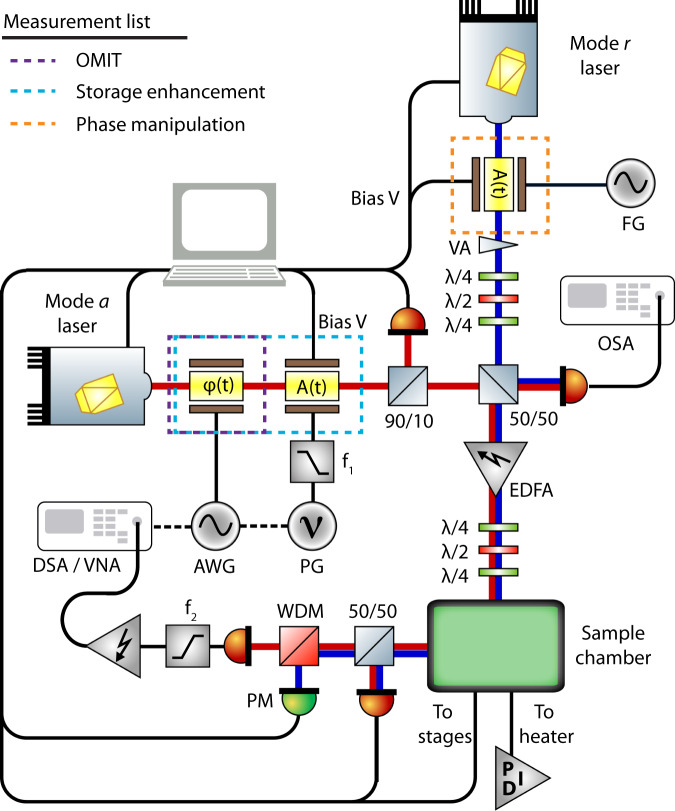
Experimental apparatus. Measurement setup including the components necessary for all measurements described in the main text. Legend indicates the optical components necessary for each measurement presented and the components are discussed in detail in the text.

The measurement apparatus for the results described in the main text is shown in Fig. 5, where the optical components added for each measurement described in the main text are outlined in the legend. Mode *a* was driven by a tunable diode laser at 1560 nm (Newport TLB-6700) while the reservoir mode, *r*, was also driven by a tunable diode laser at 1520 nm (Newport TLB-6700) whose output was connected to a variable attenuator (VA: Exfo FVA-3100). For the verification of mutual coherence, the mode *a* laser was passed through a phase electro-optic modulator (*φ*(*t*): EOSpace PM-5S5-20-PFA-PFA-UV-UL) to generate a weak probe field from the control field, which was swept across the resonance using a vector network analyzer (VNA: Keysight E5063A) allowing the measurement of OMIT. The mode *r* laser was combined with the mode *a* laser on a fiber coupled 50/50 beamsplitter (BS: Newport F-CPL-L22355-A) where one output was sent to an erbium doped fiber amplifier (EDFA: Pritel LNHPFA-30-IO) and the other to an optical spectrum analyzer (OSA: Ando AQ6317B) such that the laser wavelengths could be tracked during the experiment. The output of the EDFA was sent to a fiber polarization unit followed by the fiber taper waveguide coupled to the diamond microdisk. The output of the fiber taper was then split on another 50/50 beamsplitter where one output is sent to a power meter (PM: Newport 1936-R) and one to a 1510/1550 nm wavelength division multiplexer (WDM: Montclair MFT-MC-51-30 AFC/AFC-1) to separate the light from modes *a* and *r*. The output of the WDM was then measured on a high-speed photodetector (New Focus 1554-B) whose output is high-pass filtered and electronically amplified before being sent to the VNA for measuring OMIT or a digital serial analyzer (DSA:Tektronix DSA70804B) for the pulse-storage measurements.

**Fig. 6:**
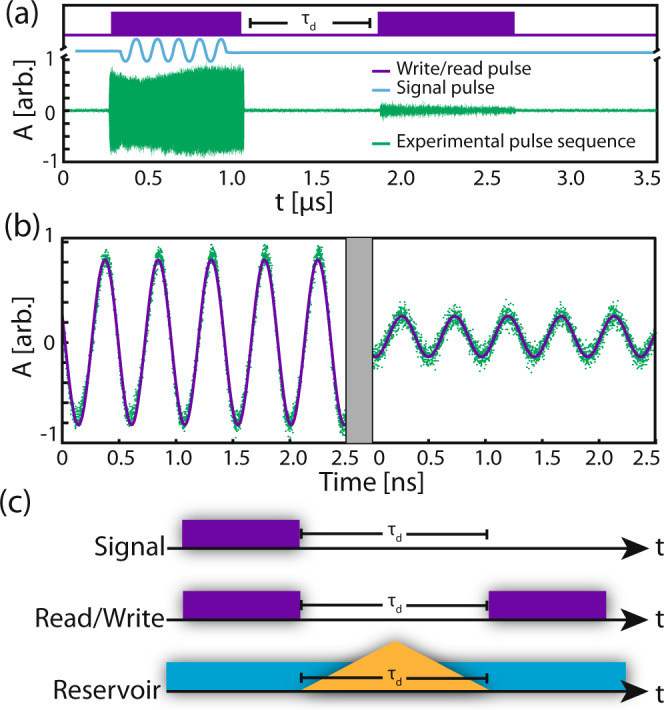
Pulse-storage measurement procedure. **a** Timing diagram for optomechanical pulse storage, along with an example of a typical measure signal (purple). **b** Detail of a short section of the write (left panel) and read pulse (right panel). **c** Timing diagram for the storage enhancement and phase-manipulation experiments described in the main text.

In the pulse-storage enhancement measurement an amplitude modulator (*A*(*t*): EOSpace AZ-0K5-10-PFA-SFA) was added to the mode *a* laser to generate the optical pulses, while the phase modulator was used to generate the signal to be written. The signal to be written was a sine wave at *ω*_b_ generated by an arbitrary waveform generator (AWG: Tektronix AWG70002A) which was superimposed on the optical pulses generated by a low pass filtered pulse generator (PG: Stanford Research Systems DG535), triggered by the AWG. Here, the reservoir-mode control laser was operated in c.w. mode, however, during the phase-manipulation measurement an amplitude modulator (*A*(*t*): Lucent Technologies 2623CS) is added to the output of the laser, which was driven by a separate function generator to generate the phase-manipulation pulses, as shown in Fig. 6. A thermoelectric heater/cooler was also placed under the sample and controlled with a PID for thermal stability during the measurement.

### Mode characterization

**Fig. 7:**
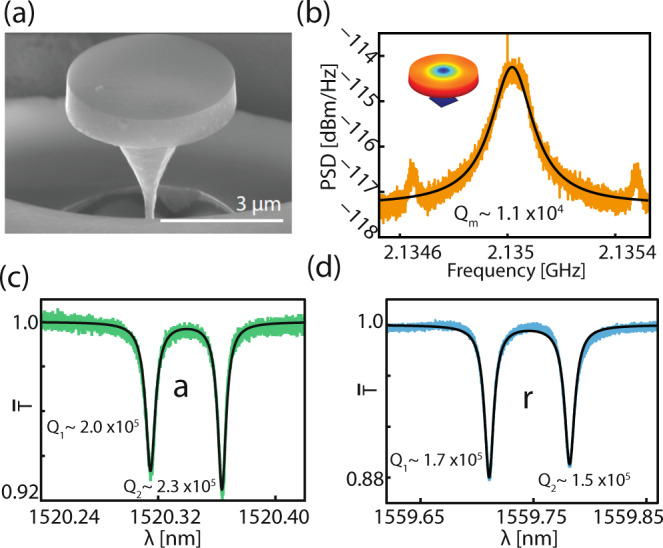
Characterization of optical and mechanical modes. **a** Scanning electron micrograph of the diamond microdisk used in this work. **b** Measured power spectral density of radial breathing mode at room temperature and pressure (inset: COMSOL simulated displacement profile). **c**, **d** Fiber taper transmission scans for both optical modes used in this work, revealing the doublet nature of the modes. Intrinsic optical quality factors are labeled.

The power spectral density for the radial breathing mode was measured using a high-speed photodetector and real time spectrum analyzer (Tektronix RSA5106A), and is shown in Fig. 7b, which was carried out at low input power (*P*_in_ ~ 50 μW) to avoid optomechanical backaction. By fitting the power spectral density to a Lorentzian, we extract a mechanical quality factor, *Q*_m_ ~ 1.1 × 10^4^, at room temperature and pressure. Optical transmission scans are shown in Fig. 7c, d, with intrinsic quality factors labeled for each of the doublet modes. The per-photon optomechanical coupling rates were measured in a separate experiment, yielding *g*_r_/*α*_r_, *g*_a_/*α*_a_ ~ 2*π* × 25 kHz, where *α*_r_ and *α*_a_ are the strong control laser amplitudes for mode *r*, and *a*, respectively (see Supplementary Note 1).

In the limit that surface scattering effects are smaller than all other optical loss mechanisms (*g*_ss_ ≪ *κ*), microdisks will possess degenerate clockwise and anticlockwise propagating modes, with negligible coupling between them. However, when the surface scattering approaches or exceeds the optical linewidth (*g*_ss_ ≥ *κ*), the clockwise and counter clockwise modes couple and will form pairs of modes known as optical doublets. These are simply symmetric and antisymmetric combinations of the traveling wave modes. The orthogonality of the doublet modes allow us to calculate the overall mechanical frequency shift, or damping rate induced by a strong control laser by taking the sum of the contributions from the symmetric and antisymmetric mode.

## Supplementary information

Supplementary Information

## Data Availability

The datasets generated during and/or analyzed during this study are available from the corresponding author on reasonable request.
