# Supplementary material for: Processing light with an optically tunable mechanical memory
Source: Nat Commun. 2021 Jan 28;12:663. doi: 10.1038/s41467-021-20899-w (PMC7844031; doi:10.1038/s41467-021-20899-w)
Supplement: Supplementary file 1 — Supplementary Information [file 41467_2021_20899_MOESM1_ESM.pdf]

Processing light with an optically tunable mechanical memory

David P. Lake, Matthew Mitchell, Denis D. Sukachev, and Paul. E. Barclay
Department of Physics and Astronomy and Institute for Quantum Science and Technology, University of Calgary, Calgary, AB, T2N 1N4, Canada[a)]

(Dated: 9 December 2020)

This document provides supplementary material for the manuscript "Processing telecom wavelength light with an optically tunable memory".

SUPPLEMENTARY NOTE 1. TUNING THE SYSTEM DYNAMICS

The results of this work are enabled by dynamic manipulation of a mechanical oscillator through engineering the degrees of freedom it is coupled to. The manifestation of this coupling is optomechanical damping, and the optomechanical spring shift, which have been extensively studied in the past[1,2]. These previous approaches leveraged frequency domain calculations and a Kubo formula to calculate damping rates, and the minimum phonon occupation. The frequency shift was then calculated through the Kramers Kronig relations. In our approach, we directly solve expressions in the time domain in the form of retarded Green's functions, and then adiabatically eliminate the fast dynamics of the cavity.

In the absence of optomechanical coupling, the mechanical mode is modelled as a damped harmonic oscillator, with annihilation operator $\hat{b}$, intrinsic frequency ω_{b}, and intrinsic damping rate Γ_{b}. The damping is a consequence of coupling to the environment, and according to the fluctuation-dissipation theorem this damping will be accompanied by a dissipation term. In the input-output formalism, we write

$$\dot{\hat{b}} = -\left(i\omega_{\mathrm{b}} + \frac{\Gamma_{\mathrm{b}}}{2}\right)\hat{b} + \sqrt{\Gamma_{\mathrm{b}}}\hat{e}_{\mathrm{in}}, \tag{1}$$

where $\hat{e}_{\mathrm{in}}$ is the input from the environment. ω_{b} and Γ_{b} are intrinsic properties of the device, however cavity optomechanics offers a way to manipulate the mechanical parameters of the cavity by optical means[3]. As we will show here, this can be viewed as coupling the mechanical mode to an optical reservoir mode, $\hat{r}$. Not only is this interaction easily controllable, but the reservoir can be arranged to have negligible thermal occupation through use of optical laser light.

We consider a reservoir mode with frequency ω_{r} connected to an input port at a rate $\kappa_{\mathrm{r}}^{\mathrm{ex}}$ with a total decay rate κ_{r}. This is dissipatively coupled to the mechanical mode with a strength g_{r}. In the presence of a strong control laser with amplitude α_{r}, the optomechanical interaction can be linearized, and we can write expressions for the cavity fluctuation operator $\hat{r}$, which is coupled the mechanics at a rate $g_{\mathrm{r}} = G_{\mathrm{r}}\alpha_{\mathrm{r}}x_{\mathrm{o}}$, where $G_{\mathrm{r}} = \frac{d\omega}{dx}$ is the shift in cavity frequency due mechanical displacement, and x_{o} are the zero point fluctuations of the mechanics[3]. This leads to the coupled equations of motion

$$\begin{bmatrix} \left(\frac{d}{dt} + \frac{1}{\chi_{\mathrm{b}}}\right) & 0 & ig_{\mathrm{r}} & ig_{\mathrm{r}} \\ 0 & \left(\frac{d}{dt} + \frac{1}{\chi_{\mathrm{b}^\dagger}}\right) & -ig_{\mathrm{r}} & -ig_{\mathrm{r}} \\ ig_{\mathrm{r}} & ig_{\mathrm{r}} & \left(\frac{d}{dt} + \frac{1}{\chi_{\mathrm{r}}}\right) & 0 \\ -ig_{\mathrm{r}} & -ig_{\mathrm{r}} & 0 & \left(\frac{d}{dt} + \frac{1}{\chi_{\mathrm{r}^\dagger}}\right) \end{bmatrix} \begin{bmatrix} \hat{b} \\ \hat{b}^\dagger \\ \hat{r} \\ \hat{r}^\dagger \end{bmatrix} = \begin{bmatrix} \sqrt{\Gamma_{\mathrm{b}}}\hat{e}_{\mathrm{in}} \\ \sqrt{\Gamma_{\mathrm{b}}}\hat{e}_{\mathrm{in}}^\dagger \\ \sqrt{\kappa_{\mathrm{r}}^{\mathrm{ex}}}\hat{r}_{\mathrm{in}} \\ \sqrt{\kappa_{\mathrm{r}}^{\mathrm{ex}}}\hat{r}_{\mathrm{in}}^\dagger \end{bmatrix}, \tag{2}$$

where we define the relevant response functions as $\chi_{\mathrm{b}}^{-1}(\omega) = \Gamma_{\mathrm{b}}/2 - i(\omega - \omega_{\mathrm{b}})$, $\chi_{\mathrm{b}^\dagger}^{-1}(\omega) = \Gamma_{\mathrm{b}}/2 - i(\omega + \omega_{\mathrm{b}})$, $\chi_{\mathrm{r}}^{-1}(\omega) = \kappa_{\mathrm{r}}/2 - i(\omega + \Delta_{\mathrm{r}})$, and $\chi_{\mathrm{r}^\dagger}^{-1}(\omega) = \kappa_{\mathrm{r}}/2 - i(\omega - \Delta_{\mathrm{r}})$. The input modes at time t, are given in terms of the time t_0 in the far past as[4]

$$\hat{e}_{\mathrm{in}}(t) = \frac{1}{\sqrt{2\pi}}\int e^{-i\omega(t-t_0)}E_0(\omega)d\omega, \tag{3}$$

$$\hat{r}_{\mathrm{in}}(t) = \frac{1}{\sqrt{2\pi}}\int e^{-i\omega(t-t_0)}R_0(\omega)d\omega, \tag{4}$$

a) Electronic mail: pbarclay@ucalgary.ca

where E_0 and R_0 are the state of the input modes at time t_0. For the sake of simplicity, in what follows, we will assume $\kappa_\mathrm{r} = \kappa_\mathrm{r}^\mathrm{ex}$.

Using Supplementary Eq. 2, we can solve for the reservoir dynamics as

$$\hat{r}(t) = \hat{r}_0 e^{-(t-t_0)/\chi_\mathrm{r}} + \int_{t_0}^{t} e^{-(t-t')/\chi_\mathrm{r}} \left(\sqrt{\kappa_\mathrm{r}}\hat{r}_\mathrm{in} + iG_\mathrm{r}\hat{b} + iG_\mathrm{r}\hat{b}^\dagger\right) dt', \tag{5}$$

$$\hat{r}^\dagger(t) = \hat{r}_0^\dagger e^{-(t-t_0)/\chi_{\mathrm{r}^\dagger}} + \int_{t_0}^{t} e^{-(t-t')/\chi_{\mathrm{r}^\dagger}} \left(\sqrt{\kappa_\mathrm{r}}\hat{r}_\mathrm{in}^\dagger - iG_\mathrm{r}\hat{b} - iG_\mathrm{r}\hat{b}^\dagger\right) dt'. \tag{6}$$

It is interesting to note the role of the optical cavity as a filter. The exponential terms are in the form of a retarded Green's function, and specify a sensitivity to frequencies near $\pm\Delta_\mathrm{r}$ to a history on the timescale $1/\kappa_\mathrm{r}$.

Inserting this into the expression for the mechanics given in Supplementary Eq. 2, we find an equation of motion for the mechanics under the influence of both the environment and the reservoir

$$\begin{aligned}\left(\frac{d}{dt} + i\omega_b\right)\hat{b} = & -\frac{\Gamma_\mathrm{b}}{2}\hat{b} - |g_\mathrm{b}|^2 \int_{t_0}^{t} \left(e^{-(t-t')/\chi_\mathrm{r}}\hat{b}(t') - e^{-(t-t')/\chi_{\mathrm{r}^\dagger}}\hat{b}(t')\right) dt' \\ & + \sqrt{\Gamma_\mathrm{b}}\hat{e}_\mathrm{in} + iG_\mathrm{b}\sqrt{\kappa_\mathrm{r}} \int_{t_0}^{t} \left(e^{-(t-t')/\chi_\mathrm{r}}\hat{r}_\mathrm{in}(t') + e^{-(t-t')/\chi_{\mathrm{r}^\dagger}}\hat{r}_\mathrm{in}^\dagger(t')\right) dt',\end{aligned} \tag{7}$$

where in the above we used the fact the $t - t_0 \gg 1/\kappa_\mathrm{r}$ and applied the rotating wave approximation. The right side of the equation can be interpreted as the sum of damping and dissipation terms due to coupling to the environment, and damping and dissipation terms due to coupling to the reservoir. With the assumption that $\kappa_\mathrm{r} \gg \Gamma_\mathrm{b}$, we can make further simplifications. First we note that the integral associated with the dissipation term becomes

$$\begin{aligned}\int_{t_0}^{t} \left(e^{-(t-t')/\chi_\mathrm{r}} + e^{-(t-t')/\chi_{\mathrm{r}^\dagger}}\right)\hat{b}(t')dt' \approx & \int_{t_0}^{t} \left(e^{-(t-t')/\chi_\mathrm{r}} - e^{-(t-t')/\chi_{\mathrm{r}^\dagger}}\right) e^{i\omega_b(t-t')}\hat{b}(t)dt' \\ = & \left(\chi_\mathrm{r}(\omega_b) - \chi_{\mathrm{r}^\dagger}(\omega_b)\right)\hat{b}(t).\end{aligned} \tag{8}$$

Next we simplify the fluctuation term as

$$\begin{aligned}&\int_{t_0}^{t} \left(e^{-(t-t')/\chi_\mathrm{r}}\hat{r}_\mathrm{in}(t') + e^{-(t-t')/\chi_{\mathrm{r}^\dagger}}\hat{r}_\mathrm{in}^\dagger(t')\right) dt' \\ &= \frac{1}{\sqrt{2\pi}} \int \int_{t_0}^{t} \left(e^{\left(\chi_\mathrm{r}^{-1} - i\omega\right)t'} e^{-\chi_\mathrm{r}^{-1}t + i\omega t_0}\hat{R}_0(\omega) + e^{\left(\chi_{\mathrm{r}^\dagger}^{-1} + i\omega\right)t'} e^{-\chi_{\mathrm{r}^\dagger}^{-1}t - i\omega t_0}\hat{R}_0^\dagger(\omega)\right) dt' d\omega \\ &= \frac{1}{\sqrt{2\pi}} \int \left(\frac{e^{(\chi_\mathrm{r}^{-1} - i\omega)t} - e^{(\chi_\mathrm{r}^{-1} - i\omega)t_0}}{\chi_\mathrm{r}^{-1} - i\omega} e^{-\chi_\mathrm{r}^{-1}t + i\omega t_0}\hat{B}_0(\omega) + \frac{e^{(\chi_{\mathrm{r}^\dagger}^{-1} + i\omega)t} - e^{(\chi_{\mathrm{r}^\dagger}^{-1} + i\omega)t_0}}{\chi_{\mathrm{r}^\dagger}^{-1} + i\omega} e^{-\chi_{\mathrm{r}^\dagger}^{-1}t - i\omega t_0}\hat{R}_0^\dagger(\omega)\right) d\omega \\ &\approx \frac{1}{\sqrt{2\pi}} \int \left(e^{-i\omega(t-t_0)}\chi_\mathrm{r}(\omega)\hat{R}_0(\omega) + e^{i\omega(t-t_0)}\chi_{\mathrm{r}^\dagger}(\omega)\hat{R}_0^\dagger(\omega)\right) d\omega \\ &\approx \frac{1}{\sqrt{2\pi}} \int \left(e^{-i\omega(t-t_0)}\chi_\mathrm{b}(\omega_\mathrm{b})\hat{R}_0(\omega) + e^{i\omega(t-t_0)}\chi_{\mathrm{r}^\dagger}(\omega_\mathrm{b})\hat{R}_0^\dagger(\omega)\right) d\omega \\ &= \chi_\mathrm{r}(\omega_\mathrm{b})\hat{r}_\mathrm{in} + \chi_{\mathrm{r}^\dagger}(\omega_b)\hat{r}_\mathrm{in}^\dagger.\end{aligned} \tag{9}$$

where once again we used the assumption that $\kappa_\mathrm{r} \gg \Gamma_\mathrm{b}$ to simplify. Combining Supplementary Eqs. 7–9 we arrive at the solution

$$\begin{aligned}\left(\frac{d}{dt} + i\omega_\mathrm{b}\right)\hat{b} = & -\left(\frac{\Gamma_\mathrm{b}}{2} + |g_\mathrm{r}|^2\chi_\mathrm{r}(\omega_\mathrm{b}) - |g_\mathrm{r}|^2\chi_{\mathrm{r}^\dagger}(\omega_\mathrm{b})\right)\hat{b} \\ & + \sqrt{\Gamma_\mathrm{b}}\hat{e}_\mathrm{in} + iG_\mathrm{r}\sqrt{\kappa_\mathrm{r}}\chi_\mathrm{r}(\omega_\mathrm{b})\hat{r}_\mathrm{in} + iG_\mathrm{r}\sqrt{\kappa_\mathrm{r}}\chi_{\mathrm{r}^\dagger}(\omega_\mathrm{b})\hat{r}_\mathrm{in}^\dagger.\end{aligned} \tag{10}$$

This can be rearranged to the simple expression reminiscent of Supplementary Eq. 1

$$\dot{\hat{b}} = -\left(i\omega_\mathrm{b}^\mathrm{eff} + \frac{\Gamma_\mathrm{b}^\mathrm{eff}}{2}\right)\hat{b} + \sqrt{\Gamma_\mathrm{b}}\hat{e}_\mathrm{in} + g_\mathrm{r}\sqrt{\kappa_\mathrm{r}}\chi_\mathrm{r}(\omega_\mathrm{b})\hat{r}_\mathrm{in} + g_\mathrm{r}\sqrt{\kappa_\mathrm{r}}\chi_{\mathrm{r}^\dagger}(\omega_\mathrm{b})\hat{r}_\mathrm{in}^\dagger. \tag{11}$$

In the above we have absorbed a factor of i into the definition of R_0 and $R_0^\dagger$, and defined effective frequency and damping terms

$$\omega_{\rm b}^{\rm eff} = \omega_{\rm b} + \omega_{\rm r}^{\rm opt} = \ \omega_{\rm b} + |g_{\rm r}|^2 \left(\frac{\omega_{\rm b} + \Delta}{\kappa_{\rm r}^2/4 + (\omega_{\rm b} + \Delta_{\rm r})^2} + \frac{\omega_{\rm b} - \Delta}{\kappa_{\rm r}^2/4 + (\omega_{\rm b} - \Delta_{\rm r})^2} \right), \tag{12}$$

$$\Gamma_{\rm b}^{\rm eff} = \Gamma_{\rm b} + \Gamma_{\rm r}^{\rm opt} = \ \Gamma_{\rm b} + |g_{\rm r}|^2 \left(\frac{\kappa_{\rm r}}{\kappa_{\rm r}^2/4 + (\omega_{\rm b} + \Delta_{\rm r})^2} - \frac{\kappa_{\rm r}}{\kappa_{\rm r}^2/4 + (\omega_{\rm b} - \Delta_{\rm r})^2} \right). \tag{13}$$

Comparing Supplementary Eq. 1 and Supplementary Eq. 11, we see that coupling the reservoir mode induces both fluctuation and dissipation. By varying the strength or detuning of the control laser, the coupling to the reservoir is modified. In the sideband resolved regime ($\omega_{\rm b} \gg \kappa$) we note two special cases. For $\Delta_{\rm r} = -\omega_{\rm b}$ the effective interaction Hamiltonian is $H_{\rm eff} = -g_{\rm r}\left(\hat{b}^\dagger \hat{r} + \hat{b}\hat{r}^\dagger\right)$, and the mechanics has the equation of motion

$$\dot{\hat{b}} = -\left(i\omega_{\rm b} + \frac{\Gamma_{\rm b}}{2} + \frac{\Gamma_{\rm r}^{\rm opt}}{2} \right) \hat{b} + \sqrt{\Gamma_{\rm b}}\hat{e}_{\rm in} + \sqrt{\Gamma_{\rm r}^{\rm opt}}\hat{r}_{\rm in}^\dagger. \tag{14}$$

On the other hand, for $\Delta_{\rm r} = \omega_{\rm b}$ the interaction Hamiltonian takes the form $H_{\rm eff} = -g_{\rm r}\left(\hat{b}\hat{r} + \hat{b}^\dagger \hat{r}^\dagger\right)$, and the equation of motion is

$$\dot{\hat{b}} = -\left(i\omega_{\rm b} + \frac{\Gamma_{\rm b}}{2} - \frac{\Gamma_{\rm r}^{\rm opt}}{2} \right) \hat{b} + \sqrt{\Gamma_{\rm b}}\hat{e}_{\rm in} + \sqrt{\Gamma_{\rm b}}\hat{r}_{\rm in}. \tag{15}$$

SUPPLEMENTARY NOTE 2. ENHANCED OMIT

The amplitude in cavity a, as a function of probe-control field detuning, $\delta_{\rm a}$, under the influence of the reservoir mode may be expressed as,

$$a(\delta_{\rm a}) = -\frac{\sqrt{\kappa_{\rm ex}}\hat{a}_{\rm in}(\omega)}{i(-\omega_{\rm b} + \delta_{\rm a}) - \kappa_{\rm a}/2 - \frac{|g_{\rm a}|^2}{i(\omega_{\rm b} + \omega_{\rm r}^{\rm opt} - \delta_{\rm a}) + (\Gamma_{\rm b} + \Gamma_{\rm r}^{\rm opt})/2}}. \tag{16}$$

From inspection with the OMIT lineshape for a conventional optomechanical system[3], when our probe is on-resonance, such that $\delta_{\rm a} = \omega_{\rm b}$, we can write our effective cooperativity as:

$$C_{\rm a}^{\rm eff} = C_{\rm a}\frac{\Gamma_{\rm b}}{\Gamma_{\rm b} + \Gamma_{\rm r}^{\rm opt}} = C_{\rm a}\frac{\Gamma_{\rm b}}{\Gamma_{\rm b}^{\rm eff}}, \tag{17}$$

where $C_{\rm a} = 4|g_{\rm a}|^2/\Gamma_{\rm b}\kappa_{\rm a}$ is the cooperativity of the device in absence of the reservoir.

A. Group delay

The group delay imparted on the pulse in transmission and reflection can be calculated about a central signal frequency, $\omega_{\rm s}$ with the spectrum confined to a small window ($< \Gamma_{\rm b}^{\rm eff}$) following Safavi-Naeini et al.[5] by computing

$$\tau^{(T)} = \mathcal{R}\left\{ \frac{-i}{t(\omega_{\rm s})}\frac{dt}{d\omega} \right\}, \tag{18}$$

and

$$\tau^{(R)} = \mathcal{R}\left\{ \frac{-i}{r(\omega_{\rm s})}\frac{dt}{d\omega} \right\}, \tag{19}$$

for the transmission and reflection group delay, respectively. These quantities are shown in Fig. 2 for both fixed and variable reservoir laser detuning.

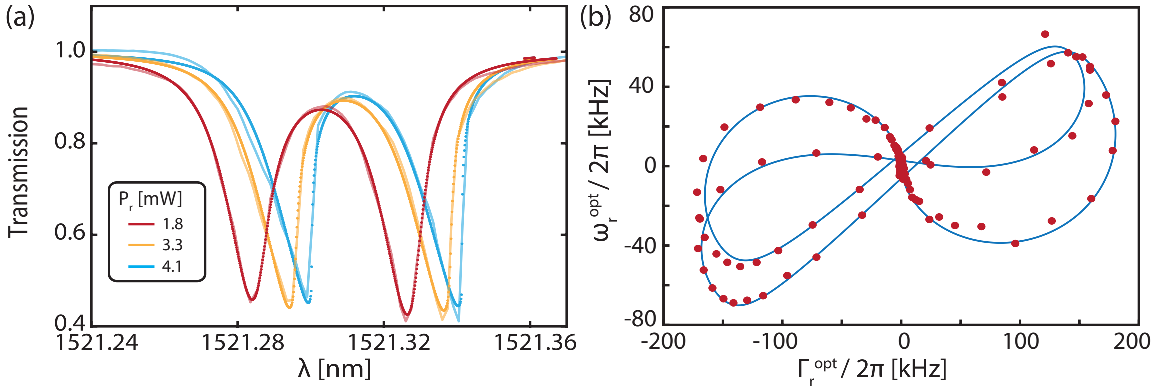

FIG. 1: (a) Optical transmission as a function of laser wavelength of the reservoir mode for increasing input power illustrating the relatively small thermo-optic shift. The $P_{\mathrm{r}} = 1.8$ mW curve corresponds to the data presented in the main text. The solid line are fits to the data taking into account thermo-optic effects in the cavity. (b) Optomechanical damping and spring effect due to mode r plotted as functions of each other, corresponding to the data shown in the main text. Here the separation of each trajectory is due to a difference in the resonance contrast of each doublet mode.

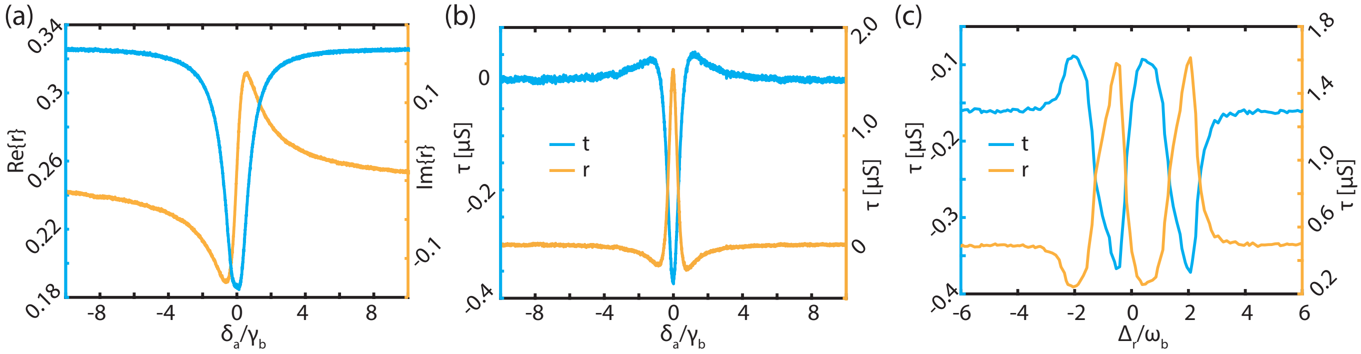

FIG. 2: (a) Real and imaginary parts of the OMIT scan corresponding to the maximum $C_{\mathrm{eff}} = 83$, where δ_{a} is the probe field detuning. (b) Extracted group delay as a function δ_{a}, for fixed control laser detuning, $\Delta_{\mathrm{a}} = \omega_{\mathrm{b}}$. (c) Extracted group delay as a function of the reservoir mode detuning, Δ_{r}, for fixed probe laser detuning, $\delta_{\mathrm{a}} = \omega_{\mathrm{b}}$.

SUPPLEMENTARY NOTE 3. COOLING AND HEATING

As a test of the reservoir engineering expressions, and as a step towards calculating the thermal occupations required for the memory calculations, we calculate full expressions for optomechanical heating and cooling here. Ignoring initial transients, the formal solution of Supplementary Eq. 11 is

$$\hat{b}(t) = \int_{t_0}^{t} e^{-\left(i\omega_{\mathrm{b}}^{\mathrm{eff}} + \frac{\Gamma_{\mathrm{b}}^{\mathrm{eff}}}{2}\right)(t-\tau)} \left(\sqrt{\Gamma_{\mathrm{b}}}\hat{e}_{\mathrm{in}} + g_{\mathrm{r}}\sqrt{\kappa_{\mathrm{r}}}\chi_{\mathrm{r}}(\omega_{\mathrm{b}})\hat{r}_{\mathrm{in}} + g_{\mathrm{r}}\sqrt{\kappa_{\mathrm{r}}}\chi_{\mathrm{r}^\dagger}(\omega_{\mathrm{b}})\right) d\tau. \tag{20}$$

We quantify the thermal statistics of the reservoir and environment with the correlators

$$\langle \hat{r}_{\mathrm{in}}^\dagger(t)\hat{r}_{\mathrm{in}}(t')\rangle = n_{\mathrm{r}}^{\mathrm{th}}\delta(t-t'), \tag{21}$$
$$\langle \hat{r}_{\mathrm{in}}(t)\hat{r}_{\mathrm{in}}^\dagger(t')\rangle = (n_{\mathrm{r}}^{\mathrm{th}}+1)\delta(t-t'), \tag{22}$$
$$\langle \hat{e}_{\mathrm{in}}^\dagger(t)\hat{e}_{\mathrm{in}}(t')\rangle = n_{\mathrm{e}}^{\mathrm{th}}\delta(t-t'), \tag{23}$$
$$\langle \hat{e}_{\mathrm{in}}(t)\hat{e}_{\mathrm{in}}^\dagger(t')\rangle = (n_{\mathrm{e}}^{\mathrm{th}}+1)\delta(t-t') \tag{24}$$

where $n_{\rm r}^{\rm th}$ is the number of thermal photons occupying the reservoir, and $n_{\rm e}^{\rm th}$ is the number of thermal phonons occupying the environment. Using these expressions, we can calculate the thermal occupancy of the cavity as

$$\begin{aligned}\langle \hat{b}^\dagger(t)\hat{b}(t)\rangle = & \int_{t_0}^{t}\int_{t_0}^{t} e^{-\left(-i\omega_{\rm b}^{\rm eff}+\frac{\Gamma_{\rm b}^{\rm eff}}{2}\right)(t-\tau)-\left(i\omega_{\rm b}^{\rm eff}+\frac{\Gamma_{\rm b}^{\rm eff}}{2}\right)(t-\tau')} \\ & \left(\sqrt{\Gamma_{\rm b}}\hat{e}_{\rm in}(\tau)+g_{\rm r}\sqrt{\kappa_{\rm r}}\chi_{\rm r}(\omega_{\rm b})\hat{r}_{\rm in}(\tau)+g_{\rm r}\sqrt{\kappa_{\rm r}}\chi_{\rm r^\dagger}\hat{r}_{\rm in}^\dagger(\tau)\right)\times \\ & \left(\sqrt{\Gamma_{\rm b}}\hat{e}_{\rm in}(\tau')+g_{\rm r}\sqrt{\kappa_{\rm r}}\chi_{\rm r}(\omega_{\rm b})\hat{r}_{\rm in}(\tau')+g_{\rm r}\sqrt{\kappa_{\rm r}}\chi_{\rm r^\dagger}\hat{r}_{\rm in}^\dagger(\tau')\right)d\tau d\tau' \end{aligned} \tag{25}$$

$$\begin{aligned} = & \int_{t_0}^{t} e^{\Gamma_{\rm b}^{\rm eff}(t-\tau)}\left(\Gamma_{\rm b}n_{\rm th,b}+\kappa_{\rm r}|g_{\rm r}\chi_{\rm r}(\omega_{\rm b})|^2 n_{\rm th,b}+\kappa_{\rm r^\dagger}|g_{\rm r}\chi_{\rm r^\dagger}(\omega_{\rm b})|^2(n_{\rm th,b}+1)\right)d\tau \\ = & \frac{\Gamma_{\rm b}n_{\rm th,b}+\kappa_{\rm r}|g_{\rm r}\chi_{\rm r}(\omega_{\rm b})|^2 n_{\rm th,b}+\kappa_{\rm r^\dagger}|g_{\rm r}\chi_{\rm r^\dagger}(\omega_{\rm b})|^2(n_{\rm th,b}+1)}{\Gamma_{\rm b}^{\rm eff}} \end{aligned} \tag{26}$$

In the experiment considered in this work, our reservoir does not have thermal occupation. Setting $n_{\rm r}^{\rm th}=0$ we recover the usual limit of optomechanical cooling

$$\langle \hat{n}\rangle = \frac{\Gamma_{\rm b}n_{\rm e}^{\rm th}+\Gamma_{\rm opt}^{\rm r}n^{\rm min}}{\Gamma_{\rm b}+\Gamma_{\rm opt}^{\rm r}} \tag{27}$$

where, $n^{\rm min}=|g_{\rm r}|^2\kappa\chi_{\rm r^\dagger}(\omega_{\rm b})/\Gamma_{\rm r}^{\rm opt}$.

SUPPLEMENTARY NOTE 4. STORAGE ENHANCEMENT

Solving the equations of motion explicitly, we can divide the phonon population in the cavity during the storage time into signal phonons, which are proportional to $\hat{a}_{\rm in}$, and undesired thermal phonons, which are a consequence of $\hat{e}_{\rm in}$. These each evolve as,

$$\langle \hat{b}_{\rm s}^\dagger(t)\hat{b}_{\rm s}(t)\rangle = \langle \hat{b_{\rm s}}^\dagger(0)\hat{b}_{\rm s}(0)\rangle e^{-\Gamma_{\rm b}^{\rm eff}t} \tag{28}$$

$$\langle \hat{b}_{\rm th}^\dagger(t)\hat{b}_{\rm th}(t)\rangle = n_{\rm e}^{\rm th}\Gamma_{\rm b}\left(\frac{e^{-\Gamma_{\rm b}^{\rm eff}t}}{\Gamma_{\rm b}+\Gamma_{\rm a}^{\rm opt}}+\frac{1-e^{-\Gamma_{\rm b}^{\rm eff}t}}{\Gamma_{\rm b}+\Gamma_{\rm r}^{\rm opt}}\right). \tag{29}$$

Here the presence of $\Gamma_{\rm a}^{\rm opt}$ terms are due to optomechanical cooling by the OMIT control laser during the write step on the initial thermal population of the resonator mode. Examples of the competing growth and decay of noise and signal phonons is shown in Fig. 3(a). Defining the storage time as the moment the signal level decays to the level of the thermal phonons, we find,

$$t_s = \frac{1}{\Gamma_{\rm b}+\Gamma_{\rm r}^{\rm opt}}\ln\left(\frac{n_{\rm s}(0)}{n_{\rm th}(0)}+\frac{\Gamma_{\rm a}^{\rm opt}-\Gamma_{\rm r}^{\rm opt}}{\Gamma_{\rm b}+\Gamma_{\rm a}^{\rm opt}}\right). \tag{30}$$

This expression is used to generate the plots in Fig. 3(b-d), which analyze the performance of the memory as a function of optomechanical damping of modes a and r. Note that quantum optical noise, e.g. Stokes scattering, is not included in this analysis.

SUPPLEMENTARY NOTE 5. PHASE SHIFTING

Reservoir engineering also allows us to dynamically change the frequency of the mechanical mode. If the frequency is changed over a time interval δt, the change in phase may be expressed as,

$$\delta\phi = \int_0^{\delta t}\left(\omega_b(t)-\omega_b(0)\right)dt \tag{31}$$

For simplicity, we assume we change our mechanical frequency as a ramp function, with maximum frequency shift $\delta_{\rm b}$. Under the adibaticity requirement $1 \gg \frac{\delta\omega_{\rm b}}{\omega_{\rm b}}$. This yields the simple expression for the phase shift,

$$\delta\phi = \frac{\delta\omega_{\rm b}\delta t}{2}. \tag{32}$$

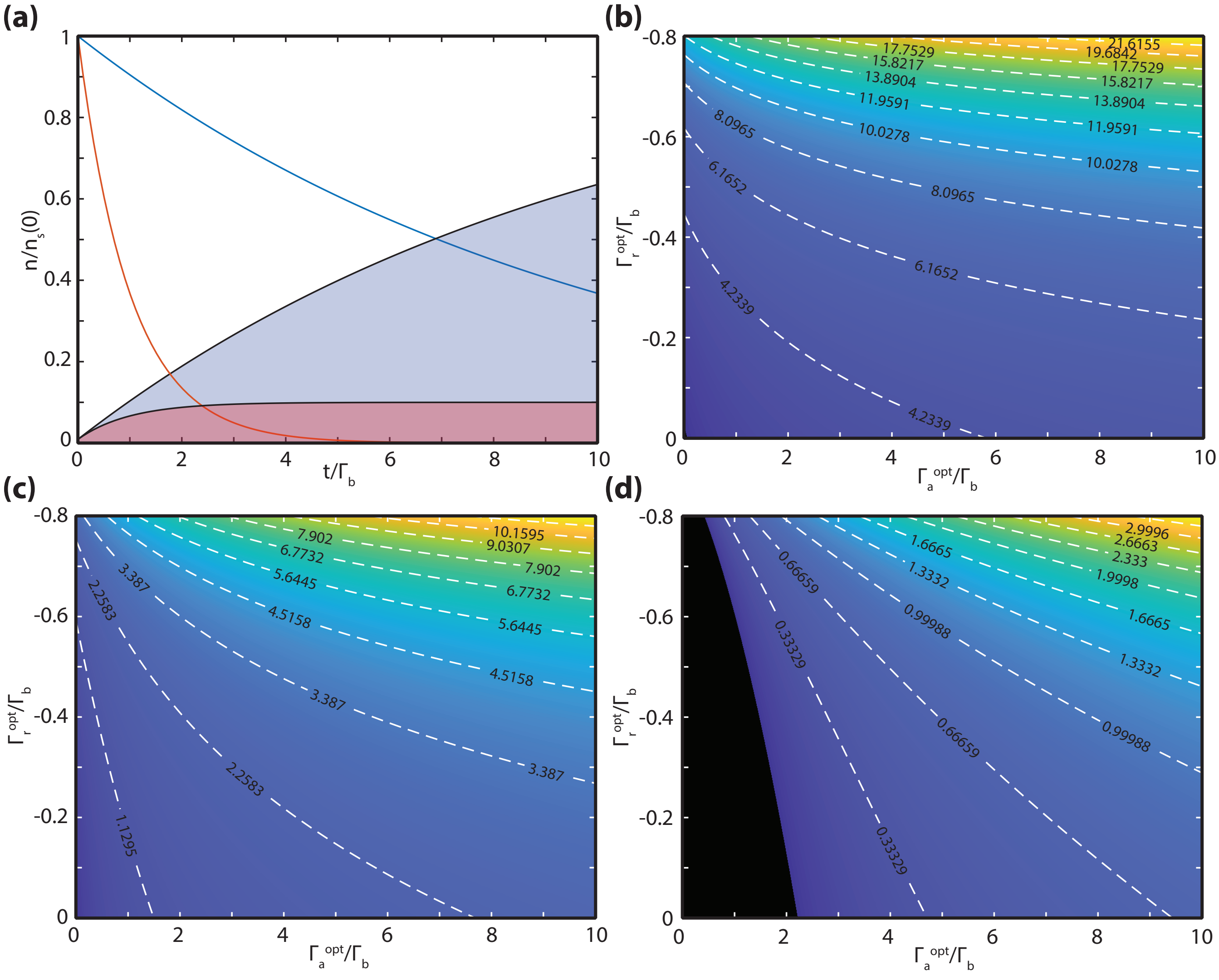

FIG. 3: (a) Signal phonons (solid lines) and thermal phonons (shaded curves) plotted for $\Gamma_{\mathrm{r}}^{\mathrm{opt}}/\Gamma_{\mathrm{b}} = 0$ (red) and $\Gamma_{\mathrm{r}}^{\mathrm{opt}}/\Gamma_{\mathrm{b}} = -0.9$ (blue). The initial signal to noise ratio $n_{\mathrm{s}}(0)/n_{\mathrm{th}}(0) = 10$, and $\Gamma_{\mathrm{a}}^{\mathrm{opt}}/\Gamma_{\mathrm{b}} = 10$. (b-d) $t_s\Gamma_{\mathrm{b}}$ vs. optomechanical damping rates for initial signal to noise ratios $n_{\mathrm{s}}(0)/n_{\mathrm{th}}(0) = \{10, 1, 0.1\}$.

In the phase shifting experiment in the main text, we operate with the reservoir laser detuning $\Delta_{\mathrm{r}} \approx -\omega_{\mathrm{b}}$, so we may approximate the frequency shift as,

$$\delta\omega_{\mathrm{b}} \approx \frac{|g_r|^2(\Delta_{\mathrm{r}} - \omega_{\mathrm{b}})}{(\Delta_{\mathrm{r}} - \omega_{\mathrm{b}})^2 + \kappa_{\mathrm{r}}^2/4}. \tag{33}$$

SUPPLEMENTARY NOTE 6. TIME LENS

The reservoir mode also permits the mechanical damping rate to be dynamically adjusted. For example, at $\Delta_{\mathrm{r}} \approx -\omega_{\mathrm{b}}$, the damping is approximately,

$$\Gamma_{\mathrm{r}}^{\mathrm{opt}} \approx \frac{-\kappa_{\mathrm{r}}|g_{\mathrm{r}}|^2/2}{(\Delta_{\mathrm{r}} - \omega_{\mathrm{b}})^2 + \kappa_{\mathrm{r}}^2/4}. \tag{34}$$

If we ramp the mechanical damping according the expression $\Gamma_{\mathrm{r}}^{\mathrm{opt}}(t) = \eta t$, we recover the expression for a time lens[6],

$$\langle \hat{b}_{\mathrm{s}}^{\dagger}(t)\hat{b}_{\mathrm{s}}(t)\rangle = \langle \hat{b_{\mathrm{s}}}^{\dagger}(0)\hat{b}_{\mathrm{s}}(0)\rangle e^{-(\Gamma_{\mathrm{b}}t + \eta t^2)}. \tag{35}$$

SUPPLEMENTARY REFERENCES

[1] F. Marquardt, J. P. Chen, A. A. Clerk, and S. M. Girvin, "Quantum theory of cavity-assisted sideband cooling of mechanical motion," Phys. Rev. Lett. **99**, 093902 (2007).

[2] I. Wilson-Rae, N. Nooshi, W. Zwerger, and T. J. Kippenberg, "Theory of ground state cooling of a mechanical oscillator using dynamical backaction," Phys. Rev. Lett. **99**, 093901 (2007).

[3] M. Aspelmeyer, T. J. Kippenberg, and F. Marquardt, "Cavity optomechanics," Rev. Mod. Phys. **86**, 1391–1452 (2014).

[4] C. W. Gardiner and P. Zoller, *Quantum Noise* (Springer, Berlin, 1991) Chap. 4, pp. 106–111.

[5] A. H. Safavi-Naeini, T. M. Alegre, J. Chan, M. Eichenfield, M. Winger, Q. Lin, J. T. Hill, D. Chang, and O. Painter, "Electromagnetically induced transparency and slow light with optomechanics," Nature **472**, 69–73 (2011).

[6] G. Patera, J. Shi, D. B. Horoshko, and M. I. Kolobov, "Quantum temporal imaging: application of a time lens to quantum optics," J. Opt. **19**, 054001 (2017).
